# T57. EFFECTS OF 0.5MS AND 1.5MS PULSE-WIDTHS ON CARDIOVASCULAR FUNCTION IN SCHIZOPHRENIA PATIENTS RECEIVING ELECTROCONVULSIVE THERAPY

**DOI:** 10.1093/schbul/sby016.333

**Published:** 2018-04-01

**Authors:** Dhruva Ithal, Sayantanava Mitra, Shyam Sundar A, C Naveen Kumar, Jagadisha Thirthalli, V J Ramesh, B N Gangadhar

**Affiliations:** National Institute of Mental Health and Neurosciences

## Abstract

**Background:**

Electroconvulsive therapy (ECT) has been shown to have a profound effect on cardiovascular functions. The initial parasympathetic response, followed by the sympathetic surge and the second parasympathetic peak characterize a typical ECT session and in patients with pre-existing cardiac disorders, this ‘roller-coaster ride’ of autonomic discharges can drastically increase morbidity and mortality; albeit such incidences are rare nowadays with the advances in medical technology. While laterality and stimulus dose (in terms of millicoulombs, mC) are known to affect cardiovascular response, the effect of pulse-width (PW) on the latter has not been explored. Compared to 1.5-milisecond (ms) stimulus pulse trains, trains with 0.5ms PW last 3 times longer for equivalent stimulus charges, other parameters remaining constant. This would translate to greater initial parasympathetic response duration, and the implications of such occurrences for cardiovascular well-being are largely unknown.

**Methods:**

Seventy-one consenting adult (M=33, F=38; mean age 30.87 ± 9.59 years, mean duration of illness 89.68 ± 77.98 months) patients, with a diagnosis of Schizophrenia, were randomly assigned to receive bilateral ECT with either 0.5ms (n=35) or 1.5ms (n=36) PW stimulus; after obtaining institutional ethical-committee’s approval. Seizure threshold was determined during the first session. Rate-Pressure product (RPP; pulse*systolic blood-pressure) was calculated during the second ECT session, in which stimulus was administered at 1.5–2 times the threshold for the two groups, at 5 time points (RPP1-5, viz. pre-anaesthesia, during anaesthesia, during convulsive motor seizure, 1 and 2 minutes post seizure, respectively). They were compared between the groups using independent-sample t-test. At baseline, the patients were assessed on PANSS for psychopathology.

**Results:**

Two groups did not differ on socio-demographic and clinical characteristics at baseline. Mean administered dose of anaesthetic agent and muscle relaxant were comparable. While the mean seizure threshold and mean charge administered at 2nd ECT were significantly lower in the 0.5 ms group, they were otherwise comparable on mean duration of seizure (motor and EEG), and the RPPs at all 5 time-points. Both Max.RPP (18102.84 ± 4477.4 mmHg/min in 0.5ms, 17935.33 ± 3598.5 mmHg/min, p=0.864) and Max.RPP-RPP2 (5010.58 ± 2893.3 mmHg/min in 0.5ms, 5811.12 ± 4270.9 mmHg/min in 1.5ms, p=0.389) were comparable between the two groups.

**Discussion:**

The characteristic sequence of cardiac events unfolding in an ECT session comprises of a temporary asystole during the administration of the stimulus, followed by an increase in blood pressure and pulse rate during clonic phase, and another slowing of heart rate at the end of motor seizure. The stimulus train duration in 0.5ms group lasts 3 times longer than in 1.5ms group for an equivalent amount of charge, thus increasing the asystole duration and theoretically altering subsequent autonomic responses. However, the groups failed to demonstrate any significant effects of these alterations in terms of altered cardiac activity implying that such alterations might not be clinically relevant. It is well known that briefer PWs cause lesser cognitive side-effects, are more efficient in eliciting seizures. present analysis shows that the two PWs of 0.5ms and 1.5ms might have similar effects on cardiovascular function, at least in otherwise-healthy adult schizophrenia patients, for similar anaesthetic agents, even if the train with 0.5ms PW lasts for double the time as with 1.5ms PW.

